# Association of culture-detected vaginal microbiota and body composition parameters with gestational diabetes outcomes

**DOI:** 10.3389/fcimb.2026.1776505

**Published:** 2026-03-23

**Authors:** Kamila Gorczyca, Żaneta Kimber-Trojnar, Małgorzata M. Kozioł, Bożena Leszczyńska-Gorzelak

**Affiliations:** 1 Department of Obstetrics and Perinatology, Medical University of Lublin, Lublin, Poland; 2 Department of Medical Microbiology, Medical University of Lublin, Lublin, Poland

**Keywords:** bioelectrical impedance, body composition, *Candida albicans*, gestational diabetes, probiotics

## Abstract

**Objective:**

To evaluate associations between culture-detected vaginal microbial colonization, body composition parameters, and lifestyle factors in women with gestational diabetes (GD).

**Design:**

Cross-sectional observational study.

**Setting:**

Tertiary university hospital, Department of Obstetrics and Perinatology, Lublin, Poland.

**Population:**

One hundred pregnant women, including 47 with GD and 53 healthy controls.

**Methods:**

Anthropometric measurements, bioelectrical impedance analysis (BIA), and vaginal culture-based microbiological assessment were performed. Dietary habits, lifestyle behaviours, and supplement use were evaluated using a validated questionnaire. BIA assessed hydration status and body composition, including total body water, extracellular and intracellular water, lean tissue mass, and body cell mass.

**Main outcome measures:**

Prevalence of selected vaginal microorganisms (*Candida albicans*, *Streptococcus agalactiae*), body composition indices, and lifestyle factors.

**Results:**

Women with GD had significantly higher BMI during pregnancy, greater lean tissue mass and body cell mass, and increased total and extracellular water compared with controls (all p < 0.05). Vaginal swabs showed a higher prevalence of *Candida albicans* (27.7% vs. 5.7%) and *Streptococcus agalactiae* (23.4% vs. 0%) in the GD group. Women with GD more frequently reported alcohol and coffee consumption before pregnancy and were less likely to use folic acid or probiotic supplements. Due to multiple comparisons, some findings may reflect type I error and should be interpreted cautiously.

**Conclusions:**

GD was associated with a higher prevalence of selected culture-detected vaginal microorganisms, as well as differences in body composition and health-related behaviours. These findings highlight potential interactions between metabolic status, microbial colonization, and lifestyle factors in pregnancy. Given the cross-sectional design, causality cannot be inferred, and comprehensive microbiome studies are needed to confirm broader ecological changes.

## 1 Introduction

Metabolic disorders are becoming increasingly prevalent worldwide. Among these, gestational diabetes (GD) represents the most common metabolic complication, affecting approximately 12–18% of pregnancies (Szmuilowicz et al., 2019; Wang et al., 2022). GD is one of the most frequently encountered conditions in obstetric practice and is associated with multiple adverse outcomes for both the mother and the fetus. Typical complications include preterm birth, preeclampsia, delivery of large-for-gestational-age (LGA) infants, shoulder dystocia, perinatal trauma, and neonatal hypoglycemia (Sweeting et al., 2022; Simmons et al., 2023).

The occurrence of GD during pregnancy serves as an important clinical warning, as it significantly increases the risk of subsequent disturbances in carbohydrate metabolism and promotes the development of cardiovascular diseases later in life, including type 2 diabetes, hypertension, and metabolic syndrome (Park and Kim, 2015; Immanuel and Simmons, 2017; Kawasaki et al., 2023).

Emerging evidence also highlights the detrimental effects of excessive sugar intake on the nervous system and brain health in later life. Elevated glucose levels are associated not only with an increased risk of postpartum depression (Žutić et al., 2024; Healy et al., 2025; Xu et al., 2025), but also with a higher likelihood of dementia and cognitive decline in the future (Pang et al., 2023; Ohene-Agyei et al., 2025).

Effective glycemic control in women with GD has been shown to reduce the incidence of macrosomia, shoulder dystocia, and preeclampsia.

Current guidelines recommend a fasting plasma glucose of ≤ 95 mg/dL (5.3 mmol/L) and 1-h and 2-h postprandial glucose targets of < 140 mg/dL and < 120 mg/dL, respectively. The HbA1c target is approximately 6.0% (42 mmol/mol), provided it can be achieved without significant hypoglycemia. Lifestyle modification, self-monitoring of blood glucose, and pharmacotherapy, when indicated, remain the cornerstone of GD management. Postpartum follow-up should include a 75-g oral glucose tolerance test (OGTT) at 4–12 weeks after delivery, followed by regular long-term monitoring (American Diabetes Association Professional Practice Committee, 2024; Amylidi-Mohr et al., 2025).

Dietary counselling emphasizing a low glycemic index and adherence to a Mediterranean-style diet has been shown to improve glycemic control, limit excessive gestational weight gain, and reduce the risk of subsequent complications (Deng et al., 2023; Di et al., 2025). Engagement in both aerobic and resistance exercise further contributes to improved glucose regulation and lowers the risk of fetal macrosomia (Martínez-Vizcaíno et al., 2023).

Pre-pregnancy body mass index (BMI) remains one of the strongest predictors of GD development. Cohort studies indicate that the risk of GD is more than doubled in overweight (BMI ≥ 25 kg/m²) and obese (BMI ≥ 30 kg/m²) women compared with those of normal weight (Zhang et al., 2021; Wu et al., 2025).

Moreover, higher BMI is associated with poorer glycemic control and a greater likelihood of requiring insulin therapy (Deng et al., 2025). Gestational weight gain also exerts a significant influence on pregnancy outcomes among women with GD. Both excessive and inadequate weight gain are linked to complications such as macrosomia, LGA or small-for-gestational-age (SGA) infants, preterm birth, and an increased rate of cesarean delivery (He et al., 2023; Perumal et al., 2023; Yang and Lai, 2024; Zheng et al., 2024; Jabin et al., 2025).

## 2 Vaginal microbiota during pregnancy

In premenopausal women, a healthy vaginal microbiota is typically characterized by low diversity and dominance by *Lactobacillus* bacteria, most commonly *L. crispatus*, and less frequently *L. jensenii*, *L. gasseri*, or *L. iners*. The dominance of *Lactobacillus* species correlates with a low vaginal pH (3.5–4.5), a lactic acid-rich environment, low inflammation, and ecological stability (Lyra et al., 2023). Protective mechanisms include lactic acid production, which has strong bactericidal effects and enhances the epithelial barrier; bacteriocins; and, to a lesser extent, hydrogen peroxide (Miko and Barakonyi, 2023; Schwecht et al., 2023). *L. crispatus* dominance is the most stable and strongly associated with protective functions (Ravel et al., 2011; France et al., 2022; Carter et al., 2023). Hormonal balance and environmental factors also influence vaginal microbiota. Estrogen stimulates epithelial maturation and glycogen deposition, with glycogen degradation products fueling lactobacilli growth, thus maintaining low pH and high lactic acid concentrations that promote stability of microbial colonization (Eslami et al., 2025). Environmental factors such as tobacco smoking exert anti-estrogenic effects, reduce glycogen availability, and disrupt *Lactobacillus* growth. Smoking further increases oxidative stress and alters vaginal metabolism by raising biogenic amines and lowering lactate levels, contributing to variations in vaginal microbial colonization (Nelson et al., 2018; Holdcroft et al., 2023). Hormonal contraception, acting through similar mechanisms, supports stable vaginal microbial colonization (Balle et al., 2023; Serrano et al., 2025). Poor intimate hygiene practices, including vaginal douching, as well as antibiotic use, can adversely affect vaginal microbial colonization patterns. Douching disrupts mucus and the normal vaginal flora, raises vaginal pH, and the surfactants and preservatives in douching products can cause microabrasions and activate local inflammatory cascades (Noyes et al., 2018; Abbe and Mitchell, 2023). Antibiotics exert short-term impacts on vaginal bacteria, including beneficial *Lactobacillus* species (Muzny and Sobel, 2023).

During pregnancy, the vaginal microbiota is usually dominated by acid-producing bacteria such as *L. crispatus*, *L. jensenii*, and *L. iners*. This leads to lactic acid production, resulting in a pH level below 4.5, and the production of antibacterial factors such as hydrogen peroxide and bacteriocins (Petricevic et al., 2014; Mendz, 2023). A robust antimicrobial barrier is created, which limits colonization of potential pathogens (Smith and Ravel, 2017). Previous studies have shown that these bacteria promote the maintenance of thick, viscous cervical mucus containing antibodies and antimicrobial peptides, which hinder bacterial ascension into the uterus, thus protecting against perinatal infections and preterm birth (Hunter et al., 2016; Lacroix et al., 2020). It has been suggested that the vaginal microbiota actively modulates the immune response through regulation of cytokines, chemokines, and complement components (Liang et al., 2024). In particular, *L. crispatus* may reduce levels of inflammatory mediators such as IL-8 and C3b/iC3b, promoting immune tolerance and lowering the risk of preterm birth, according to previous research (Gudnadottir et al., 2022; Liang et al., 2024). Maintaining low-diversity, *Lactobacillus*-dominated colonization has been associated with a decreased risk of bacterial vaginosis and preterm birth, while variations in microbial colonization may facilitate pathogen proliferation and heightened inflammation, as reported in earlier studies (Morrill et al., 2020; Chen et al., 2021; Dabee et al., 2021). Local microorganisms and their metabolites, including immunomodulatory effects observed in prior research, sustain homeostasis by protecting the vaginal mucosa from infection without provoking excessive immune activation (Cavanagh et al., 2025).

During the third trimester, maternal vaginal and intestinal microbiota demonstrate convergence, potentially preparing for childbirth. This is characterized by increased vaginal microbial diversity and decreased intestinal microbial diversity (Shin et al., 2023; Wang et al., 2023; Zhao et al., 2023; López-Moreno and Aguilera, 2024).

Previous research on the microbiota’s role in GD predominantly focused on gut microbiota and its effects on glucose metabolism and immune function. However, data on the vaginal microbiota’s potential relationship with GD development remain scarce, despite the vaginal environment’s crucial role in maintaining immune balance and infection protection during pregnancy. Moreover, the association between culture-detected vaginal microorganisms, BIA parameters, and dietary habits in women with GD has yet to be explored.

Addressing this knowledge gap could yield novel insights into GD pathogenesis and facilitate identification of biomarkers and targets for preventive interventions. Changes in body composition parameters—such as total body water (TBW), intracellular water (ICW), extracellular water (ECW), the extracellular/intracellular (E/I) ratio, lean body mass index (LMI), and body cell mass (BCM)—may influence patterns of vaginal microbial colonization by altering the metabolic and hormonal milieu of pregnant women. Lifestyle factors including diet, supplementation (e.g., folic acid and vitamin D), probiotic use, and physical activity further modulate vaginal microbial colonization.

Identifying culture-detected vaginal microorganisms in women with GD could contribute to early diagnosis of complications affecting both mother and child. Therefore, this study aimed to compare culture-detected vaginal microorganisms, anthropometric measures, and BIA results between women with GD and healthy pregnant controls, and to assess their associations with dietary and lifestyle factors, to better understand the potential role of vaginal microbial colonization as a biomarker and intervention target in GD.

## 3 Materials and methods

### 3.1 Study group

The study included 100 Caucasian women who delivered at the Department of Obstetrics and Perinatology in Lublin, Poland. Participants were divided into two groups: the first group comprised 47 women diagnosed with GD, of whom 6.7% were managed with diet alone and 93.3% received combined diet and insulin therapy. The second (control) group consisted of 53 women with uncomplicated pregnancies and no metabolic disorders. Recruitment occurred between November 2022 and January 2024. Inclusion criteria required a fasting plasma glucose level below 92 mg/dL prior to 10 weeks of gestation. In Poland, the 75-g oral glucose tolerance test (OGTT) is the standard diagnostic tool for GD. According to regulations of the Polish Ministry of Health (Lyra et al., 2023), in women without risk factors for GD—such as obesity, prior GD diagnosis, or history of macrosomia—OGTT should be performed between 24 and 26 weeks of gestation. All participants were fully informed about the study protocol and provided written informed consent. The study protocol was approved by the Bioethics Committee of the Medical University of Lublin (approval no. KE-0254/61/2020, granted on 26 March 2020). The study methodology included a medical interview and physical examination, as well as anthropometric measurements of body weight, height, waist and hip circumferences, and skinfold thickness assessed with a caliper. Body composition and hydration status were evaluated two days postpartum using bioelectrical impedance analysis (BIA). This timing was selected to standardize measurements after delivery and to avoid intrapartum hemodynamic variability; however, we acknowledge that early postpartum physiological fluid redistribution and diuresis may influence hydration-related indices. Additionally, participants completed a self-administered questionnaire comprising 50 items. This study used a culture-dependent approach to assess vaginal microorganisms and was not designed as sequencing-based microbiome profiling.

### 3.2 Laboratory testing

The material for screening tests consisted of swabs from the posterior vagina taken by a physician in accordance with aseptic principles. To assess culture-detected vaginal microorganisms, the diagnostic procedure included the following steps:

1. Microscopic evaluation of the clinical material through preparation of a direct Gram stain.
2. Culture – the swabs were streaked on culture plates using the streak plate method; standard microbiological solid media were used (enriched, selective, selective-differentiating, and special) and incubated at 37 °C for 24–72 hours under aerobic and anaerobic conditions.

Next, the bacterial and fungal colonies that grew were identified by assessing colony morphology, preparing microscopic slides, performing indirect biochemical tests, and using an automated system for species-level identification (VITEK). This culture-based approach allowed identification of selected clinically relevant cultivable microorganisms; however, it was not designed to profile the overall vaginal microbiome, determine *Lactobacillus* dominance, or define vaginal eubiosis or dysbiosis.

### 3.3 Statistical analysis

Data collected in a spreadsheet were analyzed using MedCalc 15.8 PL (MedCalc Software Ltd., Belgium) and Statistica 13 PL (TIBCO Software Inc., USA). Categorical variables were presented as absolute frequencies and percentages. Comparisons of categorical variables between groups were conducted using the chi-square test (for more than two groups/categories) or Fisher’s exact test (for two groups/categories). The distribution of continuous variables was assessed using the D’Agostino– Pearson omnibus normality test. Due to non-normal distribution of continuous variables, nonparametric tests were applied for subsequent analyses. Data were summarized using medians as measures of central tendency and interquartile ranges (IQR) and minimum–maximum values as measures of dispersion. Comparisons of continuous variables between groups were performed using the Mann–Whitney U test (for two groups/categories) or Kruskal–Wallis analysis of variance (for more than two groups/categories). Correlations between continuous variables were assessed using Spearman’s rank correlation coefficient. Statistical significance was set at p < 0.05 for all tests.

## 4 Results

A comparison was conducted between a group of patients with GD and a group of healthy controls. The groups were comparable in most demographic characteristics; however, arterial hypertension was more prevalent among women with GD (**Table 1**).

**Table 1:** Characteristics and comparison of demographic and clinical parameters between women with GD and healthy controls.

Variables	Control [n=53]	GD [n=47]	*p*
Demographic and clinical factors			
Age [years]			
Median [IQR] (min-max)	31 [24- 36] (22- 42)	28 [24-34] (22-42)	0.2632
Education			
Elementary Junior High School, Vocational Training General Secondary School Post-secondary Higher Vocational Studies Higher Education –Master’s Level	1 (1.9%) 0 (0%) 7 (13.2%) 5 (9.4%) 6 (11.3%) 14 (26.4%) 20 (37.7%)	4 (8.5%) 2 (4.3%) 8 (17%) 4 (8.5%) 4 (8.5%) 8 (17%) 17 (36.2%)	0.4324
Residence			
City over 500,000 inhabitants City with 100,000–500,000 inhabitants City with 50,000–100,000 inhabitants City with 10,000–50,000 inhabitants City under 10,000 inhabitants Village	4 (7.5%) 20 (37.7%) 6 (11.3%) 8 (15.1%) 1 (1.9%) 14 (26.4%)	3 (6,4%) 12 (2,5%) 8 (17%) 6 (12,8%) 4 (8,5%) 14 (29,8%)	0.5253
Marital status			
Married In an informal (cohabiting) relationship Single (or unmarried) Divorced/Separated Widowed	45 (84.9%) 8 (15.1%) 0 (0%) 0 (0%) 0 (0%)	37 (78.7%) 7 (14.9%) 2 (4.3%) 1 (2.1%) 0 (0%)	0.3208
Which pregnancy			
1 2 3	16 (30.2%) 30 (56.6%) 7 (13.2%)	21 (44.7%) 18 (38.3%) 8 (17%)	0. 1832
Obesity in the family			
No Yes	23 (43.4%) 30 (56.6%)	28 (59.6%) 19 (40.4%)	0.108
Diabetes in the family			
0 1 2 4 5	0 (0%) 12 (22.6%) 6 (11.3%) 29 (54.7%) 6 (11.3%)	1 (2.1%) 16 (34%) 3 (6.4%) 24 (51.1%) 3 (6.4%)	0.449
Hypertension			
No Yes	50 (94.3%) 3 (5.7%)	36 (76.6%) 11 (23.4%)	0.0111*

* statistically significant.

**Figure 1:**
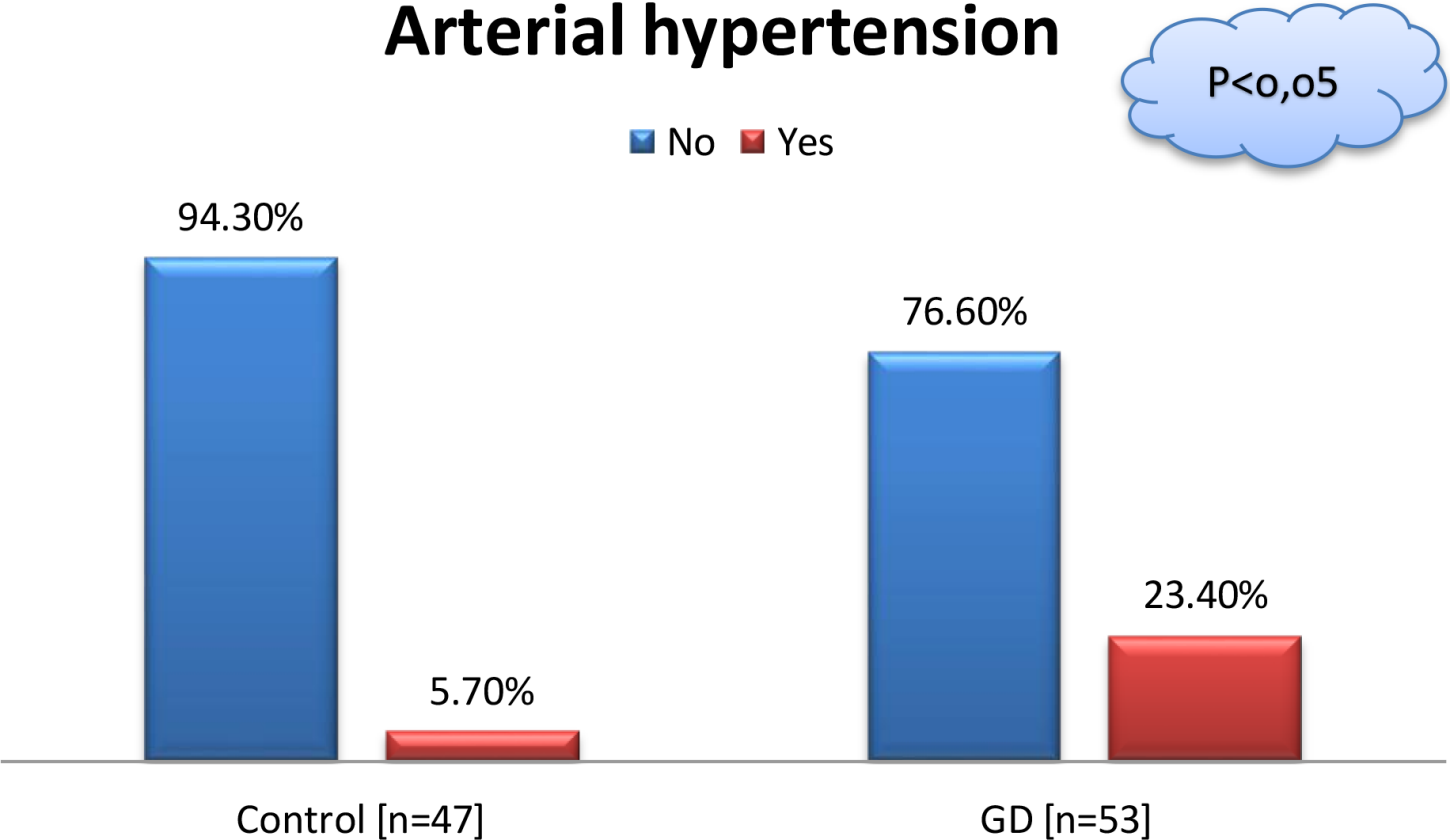
Comparison of patients with GD and controls in terms of arterial hypertension; “Chi-square test”,p < 0.05, Control n=53, GD n=47.

A significantly higher proportion of women with hypertension was observed in the GD group compared to controls (23.4% vs. 5.7%; p = 0.0111; see **Figure 1**).

Changes in the vaginal microbiota are shown in **Table 2**.

**Table 2:** Characteristics and comparison of vaginal microbiota in the group of patients with GD and controls.

Variables	Control [n=53]	GD [n=47]	*p*
Vaginal microbiota			
*Enterococcus* spp			
No	52 (98.1%)	43 (91.5%)	0.1312
Yes	1 (1.9%)	4 (8.5%)	
*Candida albicans*			
No	50 (94.3%)	34 (72.3%)	0.0029*
Yes	3 (5.7%)	13 (27.7%)	
*Klebsiella oxytoca*			
No	53 (100%)	44 (93.6%)	0.0631
Yes	0 (0%)	3 (6.4%)	
*Streptococcus agalactiae*			
No	53 (100%)	36 (76.6%)	0.0002*
Yes	0 (0%)	11 (23.4%)	
*Gram(+) Sticks*			
No	53 (100%)	46 (97.9%)	0.2883
Yes	0 (0%)	1 (2.1%)	
*Escherichia coli*			
No Yes	52 (98.1%) 1 (1.9%)	45 (95.7%) 2 (4.3%)	0.4905
*Coagulase Negative*			
*Staphylococcus*			
No	51 (96.2%)	40 (85.1%)	0.0537
Yes	2 (3.8%)	7 (14.9%)	

* statistically significant.

**Figure 2:**
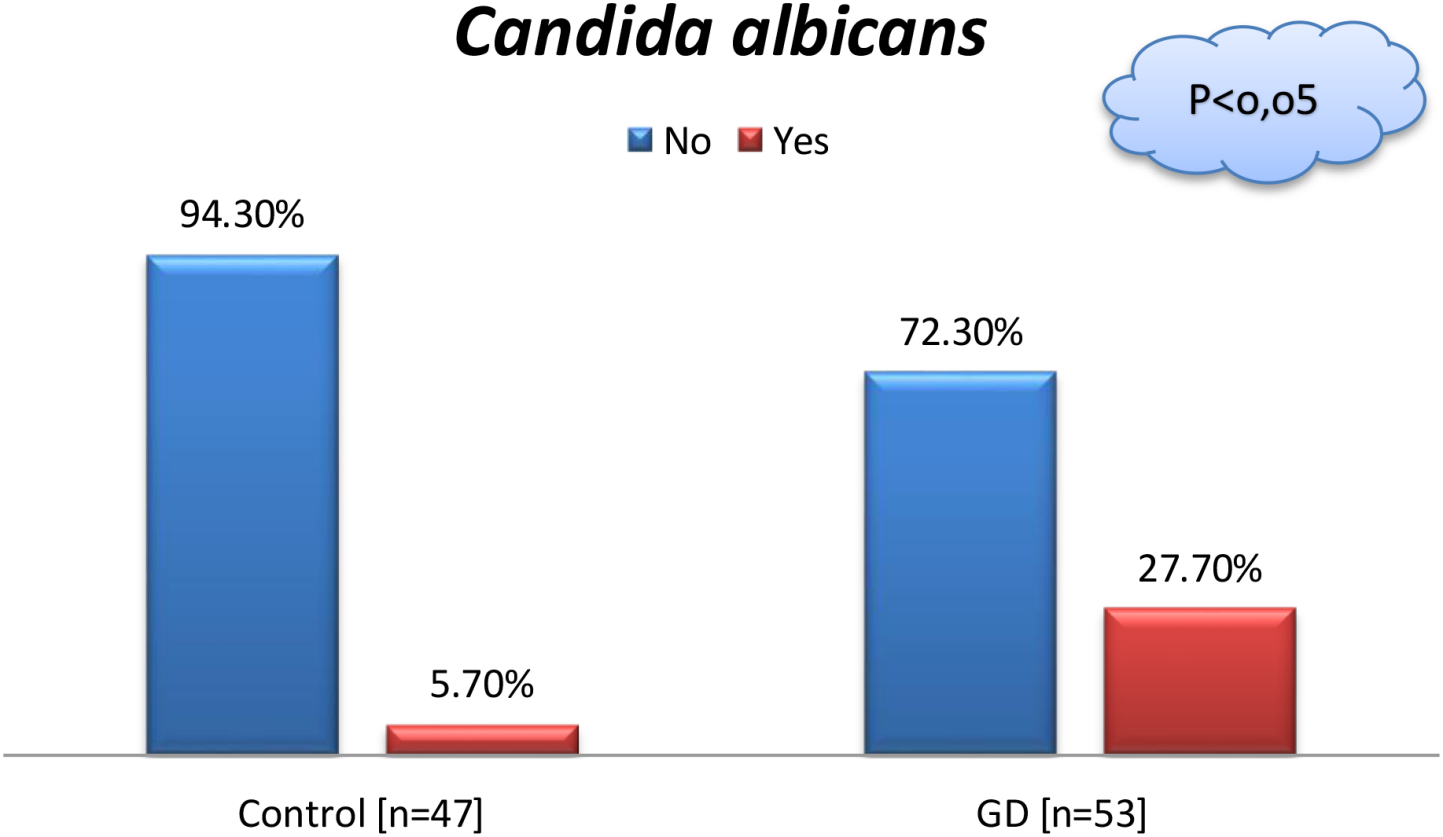
Comparison of the group of patients with GD and controls regarding the presence of *Candida albicans* in vaginal smears; “Chi-square test”,p < 0.05, Control n=53, GD n=47.

Significantly more women in the GD group tested positive for *Candida albicans* in vaginal smears compared to controls (27.7% vs 5.7%; p = 0.0029; see **Figure 2**). Similarly, *Streptococcus agalactiae* was detected more frequently in the GD group (23.4% vs 0%; p = 0.0002).

Women with GD had higher BMI and skinfold thickness compared to controls. Bioelectrical impedance analysis revealed significantly higher values of TBW, ECW, ICW, LTI, LTM, and BCM in the GD group, while OH and the E/I ratio also differed significantly between groups (**Table 3**).

**Table 3:** Characteristics and comparison of anthropometric parameters and bioelectrical impedance analysis results in the group of patients with GD and controls.

Variables	Control [n=53]	GD [n=47]	*p*
Anthropometric parameters and bioelectrical impedance analysis			
Which BMI before pregnancy			
Median [IQR] (min-max)	23,5 [22- 26.80] (18- 37.10)	25 [21.03- 29] (17.8- 36)	0.4187
Weight gain during pregnancy in kg			
Median [IQR] (min-max)	15 [10- 20] (10- 25)	15 [10.50- 18] (10- 25)	0.1059
BMI in pregnancy			
Median [IQR] (min-max)	24.70 [23.00-27.35] (16.00-33.80)	26.40 [25.30-29.30] (20.00-35.90)	0.0053*
Fold gauge			
Median [IQR] (min-max)	19.00 [10.00-22.00] (5.00-30.00)	22.00 [15.00-29.00] (7.00-38.00)	0.0007*
Waist			
Median [IQR] (min-max)	88.00 [78.00-93.00] (65.00-116.00)	90.00 [85.00-92.00] (65.00-92.00)	0.2413
Hips			
Median [IQR] (min-max)	105.00 [92.00-111.00] (78.00-128.00)	107.00 [97.50-114.00] (78.00-123.00)	0.0504
OH			
Median [IQR] (min-max)	0.60 [0.07-1.40] (-3.00-2.50)	0.20 [-1.00-1.40] (-3.40-2.50)	0.0264*
TBW			
Median [IQR] (min-max)	31.60 [26.80-33.80] (24.10-61.20)	34.90 [30.53-48.00] (24.80-48.00)	0.0004*
ECW			
Median [IQR] (min-max)	14.90 [13.00-16.05] (10.20-21.70)	16.10 [14.35-16.50] (11.30-20.20)	0.0117*
ICW			
Median [IQR] (min-max)	16.30 [14.83-17.80] (13.00-39.50)	18.60 [16.25-21.88] (13.50-25.60)	0.0003*
E/I			
Median [IQR] (min-max)	0.86 [0.81-0.96] (0.55-1.05)	0.83 [0.77-0.90] (0.57-0.97)	0.0264*
LTI			
Median [IQR] (min-max)	11.70 [10.50-12.80] (8.60-31.50)	13.50 [10.80-15.95] (10.00-18.70)	0.0037*
FTI			
Median [IQR] (min-max)	13.10 [9.78-15.33] (4.10-20.70)	13.10 [10.85-14.70] (6.50-21.60)	0.6558
LTM			
Median [IQR] (min-max)	31.50 [28.70- 35.08] (23.20- 51.10)	38.10 [31.20- 44.70] (25.60- 56.70)	0.0005*
Fat			
Median [IQR] (min-max)	26.10 [20.90-30.33] (8.70-26.50)	26.70 [22.30-29.18] (13.70-48.50)	0.5203
ATM			
Median [IQR] (min-max)	35.40 [28.38- 41.23] (11.90- 63.30)	36.30 [30.40- 39.63] (18.60- 66)	0.5781
BCM			
Median [IQR] (min-max)	16.70 [14.70- 18.50] (11- 58.90)	21 [16.20- 26.25] (13.10- 36.30)	0.0011*

* statistically significant.

Women with GD had higher BMI and skinfold thickness compared to controls. In bioelectrical impedance analysis, significantly higher values of TBW, ECW, ICW, LTI, LTM and BCM were observed in the GD group, while OH and E/I ratio differed significantly between groups (**Table 3**).

Most lifestyle variables did not differ significantly between the groups (**Table 4**). However, women in the GD group more frequently reported regular alcohol consumption in the three months preceding pregnancy compared to controls (46.8% vs. 24.5%; p = 0.0113). A higher proportion of GD patients also reported consuming coffee several times per day (25.5% vs. 9.4%; p = 0.0451). In contrast, folic acid supplementation (66% vs. 90.6%; p = 0.0027) and probiotic use (42.6% vs. 62.3%; p = 0.0499) were less common in the GD group.

**Table 4:** Characteristics and comparison of physical activity and diet in patients with GD and the control group.

Variables	Control [n=53]	GD [n=47]	*p*
Physical activity and diet			
**Physical activity before pregnancy** No Yes	35 (66%) 18 (34%)	25 (53.2%) 22 (34%)	0.1929
**Physical activity during pregnancy** No Yes	32 (60.4%) 21 (39.6%)	23 (48.9%) 24 (51.1%)	0.2534
**Frequency of activity** Inactivity Daily 2–3 times a week, Three times, but more than 30 minutes, Less than 3 times	36 (67.9%) 1 (1.9%) 2 (3.8%) 11 (20.8%) 3 (5.7%)	23 (48.9%) 2 (4.3%) 8 (17%) 13 (27.7%) 1 (2.1%)	0.1060
**Smoking cigarettes before pregnancy** 21 cigarettes and more, 11–20 cigarettes, 6–10 cigarettes, 1–5 cigarettes, Not at all	0 (0%) 0 (0%) 0 (0%) 6 (11.3%) 47 (88.7%)	0 (0%) 0 (0%) 0 (0%) 2 (4.3%) 45 (95.7%)	0.1959
**Smoking cigarettes during pregnancy** 21 cigarettes and more, 11–20 cigarettes 6–10 cigarettes 1–5 cigarettes Not at all	0 (0%) 0 (0%) 0 (0%) 0 (0%) 53 (100%)	0 (0%) 0 (0%) 0 (0%) 2 (4.3%) 45 (95%)	0.1312
**Passive smoking** No Yes	44 (83%) 9 (17%)	33 (70.2%) 14 (29.8%)	0.1307
**How often did you drink alcohol 3 months before pregnancy?** 1 2 3 4 5	1 (1.9%) 6 (11.3%) 10 (18.9%) 23 (43.4%) 13 (24.5%)	0 (0%) 1 (2.1%) 8 (17%) 16 (34%) 22 (46.8%)	0.0113*
**Type of alcohol** None, Low-alcohol beer, Beer, Beer, red wine Beer, red wine, white wine Beer, fortified wine Red wine, Red wine, white wine Red wine, strong alcohol White wine, Fortified wine, Coolers, Strong alcohols	18 (34%) 1 (1.9%) 0 (0%) 7 13.2%) 0 (0%) 4 (7.5%) 8 (15.1%) 6 (11.3%) 1 (1.9%) 2 (3.8%) 1 (1.9%) 3 (5.7%) 2 (3.8%)	18 (38.3%) 1 (2.1%) 1 (2.1%) 7 (14.9%) 2 (4.3%) 5 (10.6%) 2 (4.3%) 1 (2.1%) 4 (8.5%) 1 (2.1%) 1 (2.1%) 3 (6.4%) 1 (2.1%)	0.8648
**Avoiding the consumption of red meat (beef, pork)** No Yes	44 (83%) 9 (17%)	41 (87.2%) 6 (12.8%)	0.558
**Eating fish more often** No Yes	35 (66%) 18 (34%)	34 (72.3%) 13 (27.7%)	0.499
**Eating fruits and vegetables more often** No Yes	34 (64.2%) 19 (35.8%)	31 (66%) 16 (34%)	0.851
**More frequent consumption of milk and its products (kefir, cheese, yogurt) and eggs** No Yes	40 (75.5%) 13 (25.5%)	33 (70.2%) 14 (29.8%)	0.5564
**Number of liters of fluids per day** under 0.5 L, from 0.5–1 L, from 1–2 L, over 2 L	1 (1.9%) 18 (34%) 19 (35.8%) 15 (28.3%)	6 (12.8%) 14 (29.8%) 19 (40.4%) 8 (17%)	0.1022
**Sweets** several times a day, several times a week once a week, once a month, not at all	10 (18.9%) 21 (39.6%) 7 (13.2%) 6 (11.3%) 9 (17%)	8 (17%) 23 (48.9%) 6 (12.8%) 8 (17%) 2 (4.3%)	0.3098
**Salads** several times a day, several times a week, once a week, once a month, not at all	21 (39.6%) 27 (50.9%) 3 (5.7%) 2 (3.8%) 0 (0%)	11 (23.4%) 18 (38.3%) 7 (14.9%) 9 (19.1%) 2 (4.3%)	0.0130
**Fruit** several times a day, several times a week, once a week, once a month, not at all	39 (73.6%) 14 (26.4%) 0 (0%) 0 (0%) 0 (0%)	30 (63.8%) 7 (14.9%) 0 (0%) 6 (12.8%) 4 (8.5%)	0.0042
**Fishes** several times a day, several times a week, once a week, once a month, not at all	4 (7.5%) 13 (24.5%) 30 (56.6%) 5 (9.4%) 1 (1.9%)	9 (19.1%) 9 (19.1%) 21 (44.7%) 5 (10.6%) 3 (6.4%)	0.2981
**Coffee** several times a day, several times a week, once a week, once a month, not at all	5 (9.4%) 10 (18.9%) 4 (7.5%) 14 (26.4%) 20 (37.7%)	12 (25.5%) 12 (25.5%) 1 (2.1%) 7 (14.9%) 15 (31.9%)	0.0451*
**Do you follow a diet** No Yes *No data n=6*	17 (34.7%) 32 (65.3%)	15 (30.1%) 34 (69.9%)	0.1842
**Number of meals during the day** 0 1 2 3 4 5	0 (0%) 9 (17%) 30 (56.6%) 5 (9.4%) 8 (15.1%) 1 (1.9%)	2 (4.3%) 6 (12.8%) 32 (68.1%) 2 (4.3%) 3 (6.4%) 2 (4.3%)	0.2855
**Last meal time** 1- before 6 p.m., 2- between 6 and 10 p.m. 3- after 10 p.m.	11 (20.8%) 30 (56.6%) 12 (22.6%)	5 (10.6%) 27 (57.4%) 15 (31.9%)	0.3027
**Folic acid** No Yes	5 (9.4%) 48 (90.6%)	16 (34%) 31 (66%)	0.0027*
**Probiotics** No Yes	20 (37.7%) 33 (62.3%)	27 (57.4%) 20 (42.6%)	0.0499*
**Vitamin D** No Yes	13 (24.5%) 40 (75.5%)	20 (42.6%) 27 (57.4%)	0.0570
**Iron** No Yes	23 (43.4%) 30 (56.6%)	20 (42.6%) 27 (57.4%)	0.9326
**Omega** No Yes	21 (39.6%) 32 (60.4%)	19 (40.4%) 28 (59.6%)	0.9351

* statistically significant.

## 5 Discussion

This study indicates that women with GD exhibit differences in vaginal microbial colonization patterns, body composition parameters, and health behaviours compared with healthy pregnant women. The markedly higher prevalence of *Candida albicans* and *Streptococcus agalactiae* in the GD group indicates increased colonization by opportunistic microorganisms and increased susceptibility to opportunistic pathogens. These findings are consistent with evidence showing that hyperglycaemia and insulin resistance may modify mucosal immunity, potentially impairing epithelial barrier function and thereby favouring microbial overgrowth. Elevated glucose levels in vaginal secretions may enhance microbial proliferation, while chronic low-grade inflammation associated with GD may further reduce Lactobacillus dominance and weaken host defence mechanisms.

Our findings should also be interpreted in the context of previous studies investigating vaginal microbiota during pregnancy. Several reports have demonstrated that pregnancy is typically characterized by reduced microbial diversity and dominance of *Lactobacillus* sp*ecies*, which provide protection against opportunistic pathogens. However, metabolic disturbances may disrupt this balance. A recent study conducted in a Polish cohort of pregnant women and published in *Diabetologia* reported differences in vaginal bacterial communities between healthy women and patients with type 1 diabetes, suggesting that glycaemic dysregulation may influence vaginal microbial patterns. These observations are consistent with our results, which show increased colonization by selected opportunistic microorganisms in women with GD [52].

Importantly, our culture-based methodology does not permit comprehensive characterization of the vaginal microbiome or formal classification of eubiosis versus dysbiosis. Therefore, our results should be interpreted as differences in colonization by selected microorganisms rather than global alterations in microbial community structure. Although our culture-based approach does not allow comprehensive microbiome characterization, the higher prevalence of *Candida albicans* and *Streptococcus agalactiae* detected in the GD group aligns with the concept that metabolic and immunological alterations during diabetes may promote colonization by potentially pathogenic species. Together, these findings support the hypothesis that disturbances in glucose homeostasis may contribute to measurable changes in vaginal microbial colonization during pregnancy.

Emerging immunological evidence provides further context for the altered vaginal microbiota observed in women with GD. Hyperglycaemia is known to impair epithelial barrier integrity and modulate both innate and adaptive immune responses by reducing neutrophil chemotaxis, altering macrophage polarization, and suppressing antimicrobial peptide production. These immune disturbances create a permissive environment for colonisation by opportunistic pathogens such as *Candida albicans* and *Streptococcus agalactiae*. At the same time, chronic low-grade inflammation characteristic of GD—driven by increased cytokines such as IL-6, TNF-α, and CRP—may disrupt Lactobacillus-mediated immunoregulation, including the modulation of IL-8, C3b/iC3b, and other complement components. Loss of Lactobacillus dominance reduces lactic acid availability, raises local pH, and weakens metabolic signalling to epithelial and immune cells, further compromising mucosal resilience. These combined immunometabolic mechanisms may explain the markedly higher prevalence of Candida and GBS colonisation in the GD group in our study, which is consistent with previous reports indicating that gestational diabetes is associated with increased risk of vaginal colonization by these microorganisms (Mercado-Evans et al., 2024; Gedefie et al., 2025; Karkov et al., 2025). This highlights a bidirectional relationship in which metabolic dysregulation promotes vaginal colonization differences, while vaginal microbial shifts may amplify inflammatory responses relevant to adverse pregnancy outcomes.

In parallel, significant differences in body composition were observed. Women with GD presented higher BMI, greater lean tissue mass, and altered fluid distribution, including increased total and extracellular water. These BIA-derived abnormalities reflect metabolic and hormonal dysregulation characteristic of GD, which may be associated with differences in vaginal microbial communities through effects on tissue hydration, mucosal turnover, and systemic inflammatory pathways. Previous studies have linked abnormal body composition patterns with increased risk of fetal macrosomia and caesarean delivery, supporting the clinical relevance of these observations.

Lifestyle and supplementation behaviours also differed between groups. Women with GD were less likely to take folic acid or probiotic [43] supplements and reported higher alcohol and coffee consumption before pregnancy. Folic acid plays a role not only in neural tube development but also in glucose metabolism and immune function, while probiotics have been shown to modulate glycaemic control and support vaginal and intestinal eubiosis. These behavioural factors may therefore interact with metabolic disturbances to further destabilise the vaginal microbiota.

The combined findings underscore a multidimensional relationship between metabolic status, body composition, lifestyle factors, and microbial ecology in pregnancy. The increased colonisation rates of *C. albicans* and *S. agalactiae* in women with GD may translate into a higher risk of perinatal complications, including neonatal infections, sepsis, and preterm birth. Integrating microbiota assessment with metabolic monitoring and personalised nutritional counselling may therefore strengthen antenatal care strategies for this population.

This study has limitations. Its cross-sectional design precludes inference of causality, and culture-based microbiological methods do not provide the taxonomic resolution achievable with sequencing techniques. Additionally, lifestyle data were obtained through self-report and are subject to recall bias. Potential confounding factors such as recent antibiotic exposure, timing and duration of probiotic supplementation, and variability in glycaemic control were not fully controlled and may have influenced vaginal microbial colonization patterns. Nevertheless, the relatively large sample size and comprehensive assessment of microbiota, BIA parameters, and behavioural factors provide a robust foundation for future work. Because culture-based methods detect only selected cultivable microorganisms, our results do not provide comprehensive characterization of the overall vaginal microbiota composition and should not be interpreted as full microbiome profiling. Therefore, the observed relationships should be interpreted as associations rather than evidence of causality.

Prospective longitudinal studies incorporating metagenomic, metabolomic, and immunological profiling are warranted to clarify causal pathways linking GD to vaginal colonization differences. Identifying microbial biomarkers and characterising their interactions with metabolic and hormonal networks may enable earlier detection of risk states and the development of targeted interventions. Ultimately, the vaginal microbiota may represent a clinically meaningful tool for stratifying risk and guiding personalised management strategies in GD.

### 5.1 Clinical implications

The findings of this study highlight the clinical relevance of integrating metabolic assessment, targeted microbiological screening, monitoring of vaginal microbial colonization, and lifestyle evaluation into the routine care of women with GD [45]. The increased colonisation with *Candida albicans* and *Streptococcus agalactiae* suggests that may warrant further investigation into whether earlier microbiological screening could be beneficial and targeted preventive strategies to reduce the risk of neonatal infections and obstetric complications.

Alterations in body composition parameters, including higher extracellular water and lean tissue mass, may support the use of bioelectrical impedance analysis as an adjunct tool for identifying patients with a more adverse metabolic profile. Additionally, the observed differences in folic acid and probiotic supplementation underscore the importance of personalised nutritional counselling aimed at improving both glycaemic control and microbial balance. Together, these clinical considerations emphasise the need for a more holistic and multidisciplinary approach to antenatal care in pregnancies complicated by GD.

## 6 Conclusions

The vaginal microbiota and its proportion in the overall microbiological environment of the reproductive system play a key role in health and disease. Differences in vaginal colonization may arise from multiple factors; not only pregnancy and hygiene habits, but also coexisting diseases can influence the growth and development of specific microorganisms. Compared to healthy pregnant women, those with GD are more likely to exhibit overgrowth of *Candida* spp. and be carriers of *Streptococcus agalactiae* (GBS), which may increase the risk of perinatal complications.

GD is also associated with adverse changes in anthropometric parameters and body composition, including higher BMI during pregnancy, increased skinfold thickness, altered fluid distribution, and changes in lean mass. Differences in supplementation and lifestyle—particularly the less frequent use of folic acid and probiotics among women with GD—may further impact vaginal metabolism and microbiota composition.

These findings underscore the need for a holistic approach to the care of pregnant women with GD, incorporating glycaemic control, microbiota monitoring, body composition assessment, and tailored supplementation and lifestyle interventions. The vaginal microbiota may serve as a potential biomarker and therapeutic target for preventive strategies in GD.

## Data Availability

The raw data supporting the conclusions of this article will be made available by the authors, without undue reservation.
